# Jerky and Biltong from the Czech Retail Market: Microbial Quality, Chemical Composition, and Other Quality Characteristics

**DOI:** 10.3390/foods14213792

**Published:** 2025-11-05

**Authors:** Helena Veselá, František Ježek, Marta Dušková, Josef Kameník, Radka Hulánková, Blanka Macharáčková, Kristýna Brodíková, Renáta Karpíšková

**Affiliations:** 1 Department of Animal Origin Food & Gastronomic Sciences, Faculty of Veterinary Hygiene and Ecology, University of Veterinary Sciences Brno, 612 42 Brno, Czech Republic; veselah@vfu.cz (H.V.); jezekf@vfu.cz (F.J.); kamenikj@vfu.cz (J.K.); hulankovar@vfu.cz (R.H.); macharackovab@vfu.cz (B.M.); renata.karpiskova@med.muni.cz (R.K.); 2 Department of Public Health, Medical Faculty, Masaryk University, 625 00 Brno, Czech Republic; kristyna.brodikova@med.muni.cz

**Keywords:** dried meat, pathogenic microorganisms, physicochemical analysis, sensory analysis

## Abstract

The aim of this study was to evaluate microbiological, physicochemical, instrumental, and sensory parameters of jerky and biltong and to identify potential associations affecting their overall quality and safety. In total, 39 samples of various types of jerky (beef, turkey, venison, pork, and chicken) and 7 samples of beef biltong were analysed. The jerky and biltong samples showed low water activity values (a_w_ < 0.800), which makes them microbially stable products that can be stored at room temperature without the risk of further bacterial growth. *Listeria monocytogenes* was not detected in any of the 92 analysed samples. From a food safety perspective, the finding of *Salmonella* Enteritidis in one chicken jerky sample was unacceptable. Total viable count (TVC) values showed high variability, with findings over 8 log CFU/g. These high TVC values indicate heavily contaminated meat used to prepare dried products, or errors in the technological process that allowed bacterial growth. Both are unacceptable from a food quality and safety perspective. This means that more attention needs to be paid to the production process by processors, as well as by competent authorities. The analyses confirmed a high average protein content (>50%) in the final products and a low average fat content (<8%). The average salt content exceeded 3.0% and there was no statistically significant difference between the samples (*p* > 0.05). Similarly, there was no difference in TBARS values (*p* > 0.05). Microbial counts (TVC, lactic acid bacteria, and *Enterobacteriaceae*) were strongly affected by water activity, which was strongly negatively correlated with dry matter and its components such as proteins and ash/NaCl.

## 1. Introduction

Snacking is becoming an increasingly important component of people’s dietary habits, with nearly a quarter of daily energy intake in the United States coming from snacking [1]. Over the 1971 to 2010, the average daily energy intake from snacks rose by 132 calories per day for men and 169 calories per day for women [2]. Currently, snacks account for 15–30% of daily energy intake and thus contribute significantly to obesity [3]. This is primarily due to the fact that many popular snacks are not only calorie-dense but also nutritionally poor [3]. Jerky and biltong are among the most popular snack items worldwide, and this popularity is mainly due to the fact that they are high in protein, low in fat, and have a long shelf life [4,5,6]. Both jerky and biltong are considered nutritionally significant snack options [7,8].

Jerky is prepared from almost any lean meat, including beef, pork, poultry, or venison [9,10], but in some regions, it can also be made from domestic yak meat [11,12] or camel meat [13]. Fish can also be used [14]. Beef, ostrich, venison, and chicken meat are commonly used in biltong production. Recently, there has been an increase in the production of pork biltong, although concerns about oxidative changes to the fat still remain among processors [15].

In general, ideal jerky is characterized by a chewy texture and an appealing appearance. These characteristics are due to the combined effect of low water content, compact texture, and pronounced anisotropy [16]. Traditionally, jerky and biltong are made from thinly sliced whole-muscle meat that has been marinated and then dried [4]. Ground or minced meats can also be used, which are then shaped into strips or stuffed into a thin casing. According to the USDA definition, jerky must have a water-to-protein ratio ≤ 0.75:1 and water activity (a_w_) ≤ 0.85. An a_w_ < 0.70 is recommended to prevent mould growth [14].

Jerky is usually dried to a_w_ 0.70–0.85 [4] to ensure stability, and this gives it a tough texture. This may present difficulties for older consumers [17], and texture modification is often considered. This can be achieved either by adjusting the drying process to be less intense (semi-dry) or by using minced meat with additives to ensure binding (restructured jerky). Many jerky products therefore consist of pressed meat as an alternative to traditional sliced whole muscles, which can be cured or uncured, smoked or unsmoked, and dried using either air or hot air [18].

The Food Safety Inspection Service (FSIS) in the USA recommends a critical a_w_ limit of 0.85 or lower for the preparation of jerky for products that are stored in an aerobic or oxygen-containing environment, provided the producer ensures the prevention of mould growth on the finished product. If the product is vacuum packed in barrier packaging (creating an anaerobic environment), then the critical a_w_ limit may be 0.91 or lower [19]. These limits are based on the growth limits for *S. aureus* in the presence and absence of oxygen [19].

Although low water content and low a_w_ values make jerky or biltong products microbiologically stable, there have been cases in the past where jerky was a vehiculum for the transmission of food-borne disease agents [20,21,22]. Heat treatment of meat and hot-air drying is a widely used method of preparing jerky [23]. This practice is popular because it is simple and reduces the presence of microorganisms with high efficiency [17]. Drying reduces the water content, which can drop below 20% [12]. Differences in jerky composition arise not only due to the drying technology but also depend on the type of meat or the type of muscle used. Thus, multiple variables influence the final product’s quality. With lifestyle changes in Central and Eastern European countries, including the Czech Republic, the consumption of ready-to-eat foods, including snacks, has increased. In the last few years, a wide range of jerky or biltong products from a number of new manufacturers has appeared in the Czech retail chains. However, there is a lack of an overview of the properties of these products in terms of chemical composition, microbiological quality in relation to food safety, and also a comparison of sensory properties with information in the scientific literature. The aim of this study was therefore to compare the quality of jerky and biltong products available in the retail market in the Czech Republic, an EU member state. Microbial quality, chemical composition, organoleptic characteristics, and instrumental colour and texture parameters were determined using laboratory analyses.

## 2. Materials and Methods

### 2.1. Samples

The product purchasing was aimed at obtaining the widest possible range of products offered in grocery retail chains and gas stations. All analysed products were manufactured in the Czech Republic. The samples were stored according to the manufacturer’s recommendations. After storage, a total of 46 samples were analysed: beef (n = 16), turkey (n = 10), venison (n = 4), pork (n = 6), and chicken (n = 3) jerky and biltong (n = 7). Each sample was purchased in two different batches (February–April 2024 and June–September 2024) for a total of 78 jerky samples and 14 beef biltong samples.

### 2.2. Microbiological Analysis

Using cultivation methods according to ISO standards, the following were determined: total microbial count (ISO 4833-1:2013) [24], lactic acid bacteria count (ISO 15214:1998) [25], *Enterobacteriaceae* count (ISO 21528-2:2017) [26], moulds count (ISO 21527-2:2008) [27], coagulase-positive staphylococci (CoPS) count (ISO 6888-1:2021) [28], *Bacillus cereus* count (ISO 7932:2004) [29], and *Clostridium perfringens* count (ISO 15213-2:2023) [30]. Additionally, the presence of *Salmonella* spp. (ISO 6579-1:2017) [31] and *Listeria monocytogenes* (ISO 11290-1:2017) [32] was assessed. Samples (10 g) were cut into pieces approximately 1 cm^2^ in size and homogenized with 90 mL of buffered peptone water (all culture media were supplied by OXOID Ltd., Hampshire, UK) using a Star Blender LB 400 homogenizer (Avantor, Inc., Radnor, PA, USA). Further tenfold dilutions were prepared as needed.

Cultivation of lactic acid bacteria (LAB) was performed under anaerobic conditions using AnaeroGen™ (OXOID). From each sample, all colonies exhibiting distinct morphological characteristics (at least five colonies from each countable Petri dish) were selected and tested for catalase and oxidase activity (Erba Lachema, s.r.o., Brno, Czech Republic); both reactions yielded negative results. For the enumeration of *Enterobacteriaceae*, five colonies from two consecutive dilutions were tested for oxidase activity, which was negative. In the determination of CoPS, typical colonies (minimum of five CFU) were selected from each countable Petri dish and subsequently streaked onto Baird-Parker agar. After incubation (37 °C for 48 h), a coagulase test was performed (Erba Lachema). CoPS isolates were confirmed by PCR using detection of the SA442 fragment specific for *S. aureus* [33]. All isolates identified as *S. aureus* were examined for the presence of the epidemiologically most relevant staphylococcal enterotoxin genes (*sea*, *seb*, *sec*, *sed*, *see*, *seg*, *seh*, *sei*, and *sej*) [34,35].

Strains of *Salmonella* spp. were serotyped using slide agglutination with commercial antisera (Denka Seiken Co., Ltd., Tokio, Japan; Bio-Rad, Laboratories, Inc., Hercules, CA, USA). Prior to serotyping with “O” and “H” antisera, autoagglutination was assessed by suspending individual *Salmonella* colonies in sterile saline solution. The final antigenic structure was determined according to the White–Kauffmann–Le Minor scheme [36].

### 2.3. Physicochemical Analysis

#### 2.3.1. pH

Samples (5 g) were homogenized at 8000 rpm (DI 25 basic, IKA^®^ WERKWE, Breisgau, Germany) in 20 mL distilled water, and the pH was measured with a WTW pH 340i pH meter (WTW GmbH, Weilheim, Germany) according to Kim et al. (2021) [17]. The pH meter was calibrated with standard buffers of pH 4, 7, and 10 prior to measurement.

#### 2.3.2. Water Activity

Jerky and biltong samples (3 g) from each treatment were cut into small pieces using sharp scissors and homogenized, put into water activity cups, and their water activities were determined with a Novasina LabMaster water activity meter (Novasina, Lachen, Switzerland) according to Kim et al. (2021) [17].

#### 2.3.3. Total and Pure Protein Content

Total protein (TP) content and pure protein (PP) content were determined according to Ježek et al. (2025) [37]. TP content was determined on a Kjeltec™ 2300 instrument (FOSS, Hillerød, Denmark) using the Kjeldahl method, during which all the nitrogen in the analysed sample is determined and a factor of 6.25 is used to convert the nitrogen content into the protein content. PP content was determined after the precipitation of non-protein N-substances with hot tannin and subsequent conversion of organic nitrogen into inorganic nitrogen on a Kjeltec™ 2300 instrument as above.

#### 2.3.4. Collagen Content

The collagen content was determined according to Ježek et al. (2025) [37]. Briefly, collagen content was measured spectrophotometrically as the amount of 4-hydroxyproline at 550 nm on a GENESYSTM 6 spectrophotometer (Thermo Electron Corporation, Rochester, NY, USA). From the corrected absorbances of the calibration series, the regression coefficients of the calibration curve (the relationship between absorbance at 550 nm and the concentration of 4-hydroxyproline in µg/mL; y = 0.0146x + 0.0042) and the correlation coefficient were calculated (R^2^ = 0.9994). A calibration graph was then constructed from the corrected absorbances of the calibration series. The concentration of 4-hydroxyproline in µg/mL was determined by substituting the measured corrected absorbance into the equation of the calibration line. The hydroxyproline content was converted into the collagen content (f = 8).

#### 2.3.5. Fat Content

The fat content was determined using a Soxtec™ 2055 instrument (FOSS). Samples weighing 3 g were left in the drier for 3 h at 135 ± 2 °C and extracted with petroleum ether in the instrument for 86 min. This was followed by drying with sea sand [38] at a temperature of 103 ± 2 °C for 24 h to determine the content of dry matter. After cooling in a desiccator, the samples were weighed and the dry matter content was calculated.

#### 2.3.6. Ash Content

Ash (A) content was determined gravimetrically by burning weighed samples in a muffle furnace (Elektro LM 212.11, VEB Elektro, Bad Frankenhausen, Germany) at 550 °C until the disappearance of black carbon particles [39].

#### 2.3.7. Sodium and Salt Content

The sodium concentration was determined to verify the salt content according to Bartáková et al. (2024) [40]. First, 6 mL of concentrated nitric acid (65% *v*/*v*) and 2 mL of hydrogen peroxide (30% *v*/*v*) were added to 0.25 g of the sample and mineralised using an Ethos SEL Microwave Labstation (Milestone, S.r.l., Sorisole, Italy) at 200 °C for 30 min. The sodium content was then measured by atomic absorption spectrometry using air-acetylene flame atomisation in a contrAA 700 atomic absorption spectrometer (Analytik Jena, AG, Jena, Germany). All samples were measured in triplicate and the values obtained were analysed using Aspect CS software, version 2.1, resulting in one final value for each batch. The Na-based salt content (in %) was calculated by applying a conversion coefficient of 2.5 in accordance with Regulation No. 1169/2011 [41].

#### 2.3.8. TBARS

Thiobarbituric acid reactive substances (TBARSs) were determined to evaluate the extent of lipid oxidation. First, 97.5 mL of distilled water and 2.5 mL of 4 N HCl were added to 10 g of minced samples and left for 2 min before being homogenised. The homogenate was placed in a flask on a distillation set and heated over a gas burner, so that 50 mL of distillate was obtained in 10 min of distillation. Following distillation, 5 mL of 15% trichloroacetic acid and 0.375% thiobarbituric acid was added to 5 mL of the distillate. The samples were heated for 35 min in a boiling water bath and left to cool, after which the absorbance was measured at 532 nm in a spectrophotometer against an appropriate blank. TBARS values were calculated by multiplying the absorbance value by 7.8. TBARS quantities were expressed as malondialdehyde (MDA) equivalents (mg/kg) [42].

### 2.4. Colour

Colour was measured according to Kim et al. (2010) [43]. Colour parameters (lightness, L*; redness, a*; yellowness, b*; chroma or colour saturation, C*; hue angle, h°) were quantified according to the CIEL*a*b* system using a spectrophotometer (Konica Minolta CM-5, Konica Minolta, Tokio, Japan). The instrument was calibrated with a D65 light source and a standard observer angle of 10°, with a measuring slit of 8 mm and the specular component excluded (SCE). Each sample was measured eight times at various points on the surface. Spectra Magic 3.61 software was used to calculate the parameters.

### 2.5. Texture and Warner–Bratzler Shear Force (WBSF)

Texture was determined using the universal testing machine Instron^®^ 5544 and Software IX Series (Instron, Norwood, MA, USA). Similar to the method used by Kim et al. (2010) [43], cross-sections of jerky samples, approximately 1.0 × 2.0 cm in size (≈2.0 cm^2^), were cut to measure the Warner–Bratzler shear force (WBSF). The cross-sections were placed at a right angle to the blade. Crosshead speed was 80 mm/min. The mean WBSF value (N) for each sample was calculated from six partial measurements.

### 2.6. Sensory Evaluation

The jerky samples were marked with a random three-digit numerical code to maintain anonymity. Sensory evaluations were carried out in a specially equipped laboratory (ISO 8589:2007) [44] at the Department of Animal Origin Food & Gastronomic Sciences VETUNI Brno. The panels comprised 10 trained evaluators (ISO 8586:2023) [45] with previous experience of at least one year in sensory evaluation of meat products. The evaluation was conducted over multiple sessions, with a maximum of six jerky samples evaluated per session. The samples were cut into 1 cm-long sections and offered to the panels to evaluate sensory characteristics using a 9-point graphic scale (1 = extremely undesirable, 9 = extremely desirable), including colour (1 = too light, 9 = too dark), odour (1 = very unpleasant, 9 = very pleasant), taste (1 = very poor, 9 = very good), saltiness (1 = very low, 9 = very high), tenderness (1 = very tough, 9 = very tender), and overall acceptance (1 = very poor, 9 = very good) (ISO 4121:2011) [46]. White bread was used as a neutral tasting bite, and the evaluators had water available as needed.

### 2.7. Statistical Analysis

The results were analysed using Statistica, v. 7.1 (StatSoft CZ, Prague, Czech Republic). Microbial counts were converted to log10 CFU/g and the values below the limit of detection of the qualitative method were set to zero (if not detected) or by the limit of detection. Results on pathogens, moulds, and *Enterobacteriaceae* family were zero-inflated and thus run through a non-parametric method for non-normal distribution (Kruskal–Wallis ANOVA). The prevalence was compared using chi-squared test employing Yates’ correction. All the other data were analysed by ANOVA followed by Tukey’s HSD test for unequal samples sizes. Variables were correlated using Pearson’s correlation coefficient. *p* values below 0.05 were considered significant.

## 3. Results and Discussion

### 3.1. Microbiological Parameters

The results of the bacteriological analyses are presented in Table 1 and Table 2. There were no statistically significant differences in TVC among the six groups. However, biltong showed significantly higher TVC (*p* = 0.021) and LAB (*p* = 0.005) levels when compared to jerky, no matter what the meat origin. Occurrence of pathogens also did not differ significantly between the groups, except for *B. cereus*, with fewer positive samples detected in turkey jerky than in beef, pork, or venison jerky (*p* = 0.011).

It is important to monitor the TVC in the production of dried meat as it affects both quality and shelf life and these TVC values are influenced by various factors such as type of meat, processing method, storage conditions, etc. Initial TVC values in dried meat range from 1.0–3.0 log CFU/g. This number can multiply depend on the type of meat as well as the methods used for packaging and storage [4,47]. Table 1 shows that the average TVC of beef, turkey, pork, and venison jerky ranged between 3.0–5.0 log CFU/g, but in some samples the TVC values exceeded 7.0 log CFU/g. Yang et. al. (2009) compared TVC values for beef and pork jerky during storage [4]. The initial values were around 2.0 to 3.0 log CFU/g. After 15 days in storage, there was a significant increase in TVC for both varieties, with a significantly lower increase for beef. Our TVC results generally agreed with those initial values for all meats except chicken jerky and biltong, indicating a low overall level of microbial contamination. Chicken jerky showed the highest TVC values, reaching up to 9.8 log CFU. In batches of beef, turkey, pork, and venison jerky, we encountered samples with TVC values below the limit of detection, while no such sample was found for chicken jerky. Thus, although we observed a difference between the types of meat, the differences were not statistically significant.

Lactic acid bacteria are a natural part of the microbiota of dried meat products. In our experiment, the highest LAB numbers were found in biltong (4.0 log CFU/g) and chicken jerky (3.6 log CFU/g), with the average being around 2.0 log CFU/g. The LAB counts indicate that these are high-quality products with long shelf life. Their potential for use as a starter culture in jerky production is currently being investigated [48,49].

Bacteria belonging to the *Enterobacteriaceae* family are considered an indicator of hygiene in the production environment [50], and we found their presence in all meats except pork and venison. The highest number (3.4 log CFU/g) was found in a sample of chicken jerky.

Moulds are often mentioned in connection with jerky as contributing to spoilage. The development of moulds depends on pH, moisture content, and water activity [51]. Moulds were found in eight of the examined samples, three of which were biltong samples. Biltong also showed the highest mould counts, with a maximum of 5.4 log CFU/g. Only pork jerky was free of any moulds.

In addition to monitoring indicator bacteria, we also looked for the presence of various pathogenic bacteria. The most important pathogens that can contaminate the product after heat treatment are *S. aureus* and *L. monocytogenes*. To prevent the growth of these pathogens during storage, it is important to reduce water activity in the product to a safe level. Based on recent recommendations, drying jerky to a water activity value ≤ 0.87 is generally considered effective. This value has been reported to be sufficient to ensure that pathogenic bacteria will not grow in a vacuum-packed product stored at room temperature [52]. However, others have pointed out possible risks even at lower water activity values. Kim et al. (2018) investigated the growth and survival of *S. aureus* in beef jerky and found that it can survive in jerky with very low water activity (0.78) and even grow at temperatures above 21 °C [21]. In contrast, there was a decrease in the number of microorganisms when stored at 19 °C. These findings indicate that to ensure product safety, jerky with a_w_ of 0.78 should be stored below 19 °C to minimize the risk of *S. aureus* growth and enrichment. Ha et al. (2019) observed a reduction in *S. aureus* populations at all storage temperatures (10 °C, 20 °C, 25 °C, 30 °C, and 35 °C) in jerky with an average water activity of 0.81 [53]. Given the wide variety of jerky products available on the market (produced from different types of meat and using diverse marinades), the growth of *Staphylococcus aureus* may vary depending on specific jerky groups with similar characteristics. In our study, we detected CoPS in 11.83% of the samples, with average counts in positive samples of around 3.0 log CFU/g and the maximum was in biltong (3.4 log CFU/g). *Staphylococcus aureus* was quantified in only one dried beef sample, with a count of 1.7 log CFU/g. *Staphylococcus aureus* present at levels exceeding the threshold of approximately 5.0 log CFU/g is capable of producing sufficient quantities of enterotoxins to cause staphylococcal gastrointestinal intoxication [54]. No genes encoding staphylococcal enterotoxins were detected in our study. The risk posed by *S. aureus* increases significantly when basic hygiene rules are not consistently followed during production, especially good hygiene during product handling, such as hand washing and disinfection. Therefore, consistent control of hygiene both among personnel and in the production environment is an essential part of preventing contamination and ensuring the microbiological safety of dried meat [55].

The environment in which food is processed is the main source of *L. monocytogenes* contamination [56]. During the production of jerky, the presence of *L. monocytogenes* was confirmed both in the raw materials as well as on food contact surfaces. On the other hand, listeria was not detected in the final products. Heat treatment, marination, and reduction of water activity seem to act as sufficient barriers against the growth and enrichment of listeria [57]. *L. monocytogenes* was not detected in any of our jerky or biltong samples, which indicates good compliance with hygiene and production practices in jerky factories.

*Salmonella* Enteritidis was identified in one chicken jerky sample, which also showed the highest number of the *Enterobacteriaceae* (3.4 log CFU/g). As jerky is a ready-to-eat meat product, inadequate monitoring of production processes poses a significant risk to public health. The jerky production process typically involves drying the meat at relatively low temperatures, which allows pathogenic bacteria such as *Salmonella* spp. to survive [58]. Some *S. enterica* serovars can survive in foods with lower water activity and are more resistant to heat treatment. There are documented cases in which individuals developed salmonellosis after consuming jerky [59,60,61]. For *Salmonella*, the raw material is indeed a source of risk, but secondary contamination is more common, with *Salmonella* being introduced from production equipment, utensils, and workers. Cross-contamination is a persistent problem in smaller operations with limited space and staff, so consistent sanitation and worker hygiene is critical [22]. Increased handling during the production of jerky has been shown to significantly increase the risk of contamination of production areas and raw materials with pathogenic microorganisms. In a study by Fernandes et al. (2017), *Salmonella* spp. was detected in 3.3% of samples taken from production surfaces and 8.6% of raw meat starting material [62]. To ensure food safety, the US FSIS recommends heating meat to 160 °F, and in the case of poultry to 165 °F, before drying, precisely for the purpose of devitalizing *Salmonella* or STEC. There is no such recommendation in European food legislation and there is no such recommendation in Czech national legislation either. Based on the results of this study, where salmonella was detected despite low a_w_ values of <0.900, the authors recommend adopting appropriate standards for jerky/biltong production [19].

The spore-forming species *B. cereus* is also associated with jerky [51]. In our study, we detected *B. cereus* in all the analysed types of jerky. The highest levels were in venison, turkey, and beef jerky. In the samples that were positive for *B. cereus*, the average count was 2.0 log CFU/g. There was a statistically significant difference in *B. cereus* counts between the different types of jerky.

*Clostridium perfringens*, another spore-forming species, was not detected by plate counting but was found only after enrichment (see Table 2). Our results support the conclusions of Nam et al. (2018), who reported that the risk of *C. perfringens* contamination in jerky is very low [63].

### 3.2. Physicochemical Parameters

The results of the physicochemical parameters of the jerky samples are presented in Table 3. A statistically significant difference was found between the pH in chicken jerky and biltong (*p* = 0.009). The lower pH of biltong was likely due to the method of meat processing and the composition of the pickling liquor. While jerky is brined in a mixture of water, salt, spices [64], or sugar, sodium nitrite, and sodium erythorbate [4], biltong brine typically contains brown spirit vinegar or apple cider vinegar at 3–6% [15]. Lim et al. (2014) found pH values for beef jerky ranging from 5.90 to 5.68, depending on marinade composition and marinating time [65]. Similar values (ranging from 5.73 to 5.64) were also found by Shi et al. (2021), and they attributed the differences to regional characteristics and processing methods that affect the pH and quality of cured meat products [64].

The lowest water activity (a_w_) was found in chicken jerky and the highest a_w_ in venison jerky, but the differences between species were not statistically significant (*p* > 0.05). Yang et al. (2009) found something similar for beef and pork jerky: the a_w_ of jerky samples varied from 0.835 to 0.794 with storage, and none of the jerky samples showed any significant a_w_ changes over 30 days of storage [4]. Water activity values can vary widely in commercially available jerky: Ingham et al. (2006) reported a_w_ for beef jerky ranging from 0.47 to 0.87 [52]. Moreover, the drying method can influence a_w_. Shi et al. (2021) found a_w_ values ranging from 0.44 to 0.53 for freeze-dried jerky, while hot air-dried jerky had a_w_ values between 0.60 and 0.70 [64]. In addition to drying, a_w_ can be reduced by the addition of salt [15].

The total protein (TP) content of chicken jerky was significantly higher (*p* = 0.029) than that of beef jerky. There was no difference among the other samples. The TP content found in this study was similar—57.8–64.3% and 57.5–66.4%—to that reported for beef jerky by Shi et al. (2021) and Cheng et al. (2023) respectively [64,66]. Drying increases the protein content, which can reach 75.5–79.1 g/100 g dry matter in beef jerky [18]. There were no significant differences in pure protein content between the samples in this study (*p* > 0.05).

Collagen is the most abundant protein in connective tissue and is responsible for the textural differences in meat. Higher collagen concentrations and mature crosslinks have an additive effect on the toughness of the meat and yield fewer tender meats [6]. Chicken, pork, and turkey jerky contained the least collagen content. Significantly higher (*p* < 0.001) collagen content was found in beef jerky and biltong.

We found differences in fat content, with turkey jerky having significantly (*p* = 0.001) lower fat than pork jerky or biltong. Chicken jerky and venison jerky also had low fat contents, but this difference was not significant (*p* > 0.05). A slightly higher fat content was found for beef jerky by Vidal et al. (2025), who reported values ranging from 9.2% to 13.9% [67].

Dry matter content in our samples ranged from 69.2% (biltong) to 76.2% (chicken jerky), but the differences between samples were not significant (*p* > 0.05). Drying reduces the water content of jerky, which can fall below 20% [12]. Shi et al. (2021) found water contents of 9.58% and 11.9% in beef jerky samples that were dried naturally over two winter months in northern China, and 15.2% and 22.2% for jerky prepared by hot-air drying [64].

Differences in jerky composition arise not only due to the drying technology but also depending on the type of meat and the specific type of muscle used. Yang et al. (2009) prepared beef jerky using the semimembranosus muscle and pork jerky using the semimembranosus, the longissimus dorsi, and the psoas major muscles taken 48 h after slaughter [4]. The proportion of water in the jerky samples ranged from 24.45% to 27.69%. These values agree with the dry matter contents found in our jerky and biltong samples in present study.

The ash content did not differ among our samples (*p* > 0.05). While Cheng et al. (2023) reported ash content in beef jerky ranging from 2.68 to 3.82% [66], Shi et al. (2021) found higher values (10.8 to 13.2%) for beef jerky [64]. The higher ash content is likely related to the lower moisture content.

There were no significant differences in salt content among the samples with values between 3.44 and 3.73%. Lim et al. (2014) reported salt content in beef jerky between 2.55 and 3.05% [65], while for biltong, Engez et al. (2012) found values ranging from 2.68 to 3.30% [68]. Salt used to marinate meat can vary from 1.5% [4] to 4% [15], and the final salt content can be influenced by the marination duration [65] as well as the method of drying [68]. Salt is essential not only as a seasoning, but also in its preservation as it has a dehydrating effect, thus helping to cure the meat. High salt concentrations in the brine can increase the salt uptake into the meat and consequently decrease the a_w_ [6].

The TBARSs value is the most common measure of the degree of lipid oxidation in meat products. Differences in mean TBARS values between the jerky and biltong samples were not significant (*p* > 0.05) and ranged from 2.13 to 3.30 mg MDA/kg. Particularly in poultry jerky, there were large differences in TBARS values, as shown in Table 3. Similar TBARS values were also found by Yang et al. (2009) for beef and pork jerky during storage [4]. It is normally accepted that the TBARSs value increases over time in the meat during storage, although the pattern of this increase in different varieties is not well known. Further, there is a proportional increase of lipid oxidation as a_w_ decreases. Particularly, TBARS values tend to be higher as drying time increases and also depend on the salt concentration [69]. Lim et al. (2014) reported the effect of curing solution composition and curing time on TBARS values, which ranged from 1.59 to 3.85 mg MDA/kg, while the concentration of Na^+^ had a significant effect on the degree of lipid oxidation [65]. Zhou et al. (2021) investigated the effect of curing time on TBARS values and found that when the fermentation time was 0–12 h, the TBARSs value increased only slowly, and given that the microbiota present had low lipolytic activity, the increase of TBARSs value was mainly attributed to biochemical conversion [70]. The levels of fatty acids produced by lipid hydrolysis were not very high, and the accumulated aldehydes mainly came from the original unsaturated fatty acids in the samples.

From all analysed samples biltong was shown to have a significantly lower pH (*p* = 0.004) and dry matter content (*p* = 0.018), together with higher collagen content (*p* < 0.001).

### 3.3. Colour and Texture Parameters

The colour parameters of the jerky and biltong samples are summarised in Table 4. Pork and turkey jerky had significantly higher (*p* = 0.040) L* compared to beef and chicken jerky samples. Biltong and chicken jerky had lower (*p* = 0.014) a* than pork and turkey jerky. C* was lowest in chicken jerky, and significantly higher C* values (*p* = 0.029) were measured in pork and turkey jerky. No differences (*p* > 0.05) were found for b* and h°. There are only a few reports comparing the colour parameters of jerky from different animal meats. Yang et al. (2009) reported that the lower L* value of beef compared to pork jerky is likely due to the higher proportion of red muscle fibres in beef muscles than in pork muscles [4]. Darkening as a result of reduced a_w_ was the only effect discernible by the eye [71]. The drying process has a significant influence on L*, a*, and b* values, which contribute to the variation in light scattering from the surface of the meat that is perceived as the degree of browning during drying [6]. The colour of the final product is also influenced by the composition of the marinating liquor. Given the same temperature, surface colour values of beef jerky can change depending on the marinade ingredients, and the L* value decreases with the use of soy sauce, red pepper paste, and soybean paste solutions [65]. Moreover, surface colour values, especially a* can increase if a bacterial starter culture is added [72].

Texture is also an important factor meat products and influences consumer preference. One of the most important attributes of jerky is also its hardness, which can be measured as shear force [65]. Our results show that the Warner–Bratzler shear force (WBSF) decreased as follows: beef jerky > biltong > pork jerky > venison jerky > turkey jerky > chicken jerky, ranging between 52.8 and 83.9 N. However, the differences were not statistically significant (*p* > 0.05). Similar values for beef jerky were also found by Lim et al. (2014) [65]. Higher firmness and toughness may be due to higher uptake of the marinating liquor resulting in the firm texture of dried jerky samples [18]. Higher WBSF values could also be related to its lower moisture content and muscle fibre composition [4].

### 3.4. Sensory Analysis Results

The most important sensory attributes of jerky (and other snack foods) are texture, colour, and flavour, which are determined by the raw material and numerous other technological factors [65]. Sensory panels were convened to assess the effects of colour, odour, taste, saltiness, tenderness, and overall acceptance of analysed samples (Table 4). There were no differences (*p* > 0.05) in odour, taste, saltiness, and overall acceptance between samples. The colour of pork, chicken, and turkey jerky was significantly different (*p* < 0.001) from venison jerky. In addition, pork and turkey jerky had lower scores than biltong and beef jerky. Differences in colour could not be assessed in a meaningful manner due to the different types of meat. Biltong was judged to be significantly more tender (*p* < 0.001) than beef, turkey, and venison jerky. The tenderness of jerky samples ranged from 3.70 to 5.36 and although venison jerky was the fattiest, the tenderness differences between the jerky varieties were not significant (*p* > 0.05). Although there was no difference in overall acceptability among samples, it was most affected by taste (R = 0.91), odour (R = 0.66), and saltiness (R = 0.48; *p* < 0.05). The soybean is an important food for the supply of essential amino acids, unsaturated fatty acids, isoflavones, phytic acid, saponin, trypsin inhibitors, tocopherol, and oligosaccharides. These substances are the origins of the unique flavours and aromas of the soy beans [65].

### 3.5. Correlation Between Parameters

Our correlation analysis revealed some interesting relationships, or, in some cases, a surprising lack of them. As could be expected, microbial counts (TVC, LAB, and *Enterobacteriaceae*) were strongly affected by water activity (*p* ≤ 0.003), which was strongly negatively correlated with dry matter (R = −0.94, *p* < 0.001) and its components, such as proteins and ash/NaCl (*p* = 0.007 and *p* < 0.001 for NaCl and other parameters, respectively). As expected, NaCl content correlated with saltiness (R = 0.28, *p* = 0.011), but increasing fat content did not automatically lead to increased values for the products of fat oxidation—TBARS (*p* = 0.519). This could be explained by differing fat composition—e.g., the ratio of saturated and unsaturated fatty acids, which makes beef fat generally less prone to oxidation in comparison to pork [4] and especially poultry [9]. Increased TBARS values significantly negatively affected taste (R = −0.38, *p* = 0.001), odour (R = −0.24, *p* = 0.036), and the overall acceptability of the products (R = −0.30, *p* = 0.009). WBSF did not correlate with tenderness (R = 0.10, *p* = 0.371) or any of the physicochemical parameters, while tenderness itself was affected by protein content (R = −0.34, *p* = 0.002), but not by collagen content (R = 0.05, *p* = 0.649). Surprisingly, we did not see any relationship between collagen and tenderness, but this is likely due to the fact that collagen content in jerky/biltong is generally very low [4].

## 4. Conclusions

This study confirmed that dried meat products in the snack category (specifically jerky and biltong) did not exceed a_w_ values of 0.900, which guarantees them high microbial stability and eliminates the need for refrigerated storage. *Listeria monocytogenes* was not detected in any of the analysed samples. The analysed samples were found to have a high protein content, a low fat content, and a low collagen content, which makes these foods a suitable supplementary source of high-quality proteins.

Despite the microbial stability of jerky and biltong, a significant part of the analysed samples showed an increased level of TVC, which exceeded 6.0 log CFU/g. The higher microbial contamination of the samples may reflect (i) high contamination of the input raw material without reduction of the number of microorganisms during the production process or (ii) inappropriately set production procedures with the possibility of cross-contamination by microorganisms.

While most of the analysed products met the contamination level comparable to excellent quality fresh meat (≤3.0 log CFU/g), one chicken jerky sample with high levels of TVC and *Enterobacteriaceae* contamination was even positive for *S*. Enteritidis. No statistically significant differences in TVC levels were found between samples from different types of meat.

The results of this study clearly demonstrate that to ensure microbial safety and high quality of dried meat snacks, it is essential for manufacturers and competent authorities to pay increased attention to the entire production chain. The key is to effectively control the microbial load of input raw materials and implement effective measures to prevent cross-contamination and ensure the reduction of the number of microorganisms during processing up to the final packaging stage. Only in this way can consumers be offered safe products without the risk of infection by foodborne pathogens.

## Figures and Tables

**Table 1:** Occurrence of total viable count (TVC), lactic acid bacteria (LAB), *Enterobacteriaceae* (*Enterobact*.), and moulds in different types of jerky/biltong. Results are presented as log CFU/g (mean log CFU/g; minimum; maximum value; number and percentage of samples positive for the presence of a group of microorganisms).

Parameter	Beef n = 32	Turkey n = 20	Pork n = 12	Venison n = 8	Chicken n = 6	Biltong n = 14
TVC	3.4 [<1.0; 8.3] 31 [96.9%]	3.0 [<1.0; 7.3] 17 [85.0%]	3.3 [<1.0; 8.3] 11 [92.7%]	2.9 [<1.0; 5.0] 7 [87.5%]	5.0 [2.0; 9.8] 6 [100.0%]	4.7 [1.85; 7.6] 14 [100.0%]
LAB	2.4 [<1.7; 7.5] 20 [62.5%]	2.5 [<1.7; 7.2] 12 [60.0%]	<1.7 * [<1.7; <1.7] 6 [50.0%]	2.7 [<1.7; 4.6] 7 [87.5%]	3.6 [<1.7; 7.3] 4 [66.6%]	4.0 [<1.7; 6.7] 12 [85.7%]
*Enterobact*.	0.6 [<1.0; 3.0] 3 [9.4%]	0.7 [<1.0; 2.04] 3 [15.0%]	<1.0 * [<1.0; <1.0] 0 [0.0%]	<1.0 * [<1.0; <1.0] 0 [0.0%]	1.3 [<1.0; 3.4] 2 [33.3%]	0.7 [<1.0; 2.6] 1 [7.1%]
Moulds	1.1 [<2.0; 2.8] 5 [15.6%]	1.1 [<2.0; 2.5] 1 [5.0%]	<2.0 * [<2.0; <2.0] 0 [0.0%]	1.4 [<2.0; 2.5] 3 [37.5%]	1.2 [<2.0; 2.3] 1 [16.6%]	1.8 [<2.0; 5.4] 2 [14.3%]

* all samples below the detection limit by plate counting method.

**Table 2:** Occurrence of *Bacillus cereus*, coagulase-positive staphylococci (CoPS), and *Clostridium perfringens* in different types of jerky/biltong. Results are presented as log CFU/g (mean log CFU/g; minimum; maximum value; number and percentage of samples positive for the presence of a group of microorganisms after enrichment).

Parameter	Beef n = 32	Turkey n = 20	Pork n = 12	Venison n = 8	Chicken n = 6	Biltong n = 14
*B. cereus*	1.1 [<1.7; 2.6] 23 ^bc^ [71.9%]	1.0 [<1.7; 2.2] 7 ^a^ [35.0%]	<1.7 * [<1.7; <1.7] 10 ^bc^ [83.3%]	1.2 [<1.7; 2.3] 7 ^bc^ [87.5%]	<1.7 * [<1.7; <1.7] 4 ^ac^ [66.7%]	1.0 [<1.7; 1.7] 6 ^ab^ [42.9%]
CoPS	0.9 ** [<1.7; 3.3] 4 [12.5%]	0.9 [<1.7; 2.0] 1 [5.0%]	1.1 [<1.7; 3.1] 1 [8.3%]	<1.7 * [<1.7; <1.7] 0 [0.0%]	<1.7 * [<1.7; <1.7] 1 [14.3%]	1.0 [<1.7; 3.4] 4 [28.6%]
*C. perfringens*	<1.7 * 2 [6.3%]	<1.7 * 0 [0.0%]	<1.7 * 0 [0.0%]	<1.7 * 1 [12.5%]	<1.7 * 0 [0.0%]	<1.7 * 1 [7.1%]

^a,b,c^ Different superscripts represent statistically significant differences between values within each row; * all samples below the detection limit by plate counting method; ** *Staphylococcus aureus* was quantified at 1.7 log CFU/g in one sample.

**Table 3:** Chemical analysis of jerky/biltong.

	Biltong	Beef Jerky	Pork Jerky	Chicken Jerky	Turkey Jerky	Venison Jerky	*p* Value
pH	5.22 ± 0.21 ^a^	5.46 ± 0.27 ^ab^	5.36 ± 0.23 ^ab^	5.76 ± 0.16 ^b^	5.52 ± 0.44 ^ab^	5.47 ± 0.19 ^ab^	0.009
a_w_	0.760 ± 0.087	0.722 ± 0.095	0.748 ± 0.111	0.682 ± 0.127	0.740 ± 0.098	0.765 ± 0.051	0.506
Total protein [%]	50.3 ± 7.1 ^ab^	50.2 ± 10.0 ^a^	54.1 ± 6.8 ^ab^	59.0 ± 9.5 ^b^	56.83 ± 9.0 ^ab^	57.2 ± 5.6 ^ab^	0.029
Pure protein [%]	43.5 ± 7.2	43.4 ± 8.6	46.8 ± 5.9	50.9 ± 5.9	48.0 ± 7.5	49.5 ± 3.8	0.054
Collagen [%]	2.29 ± 0.66 ^a^	1.95 ± 0.63 ^ab^	1.08 ± 0.24 ^c^	0.98 ± 0.18 ^c^	1.13 ± 0.32 ^c^	1.47 ± 0.63 ^bc^	<0.001
Fat [%]	7.54 ± 3.95 ^a^	6.54 ± 3.01 ^ab^	7.77 ± 3.72 ^a^	4.81 ± 0.98 ^ab^	3.92 ± 1.78 ^b^	4.71 ± 2.11 ^ab^	0.001
Dry matter [%]	69.2 ± 8.3	75.2 ± 6.5	73.3 ± 8.2	76.2 ± 9.2	73.4 ± 6.6	72.8 ± 4.0	0.196
Ash [%]	6.52 ± 1.19	6.34 ± 1.36	6.54 ± 1.35	7.41 ± 0.88	6.74 ± 1.27	6.44 ± 0.38	0.494
NaCl [%]	3.55 ± 0.74	3.53 ± 0.64	3.44 ± 0.60	3.73 ± 0.43	3.70 ± 0.71	3.63 ± 0.31	0.870
TBARS [mg MDA/kg]	2.52 ± 1.49	3.30 ± 2.72	2.13 ± 1.51	2.63 ± 4.63	3.14 ± 5.19	2.16 ± 1.46	0.540

^a,b,c^ Different superscripts represent statistically significant differences between values within each row.

**Table 4:** Colour, texture, and sensory analysis of jerky/biltong dried meat.

	Biltong	Beef Jerky	Pork Jerky	Chicken Jerky	Turkey Jerky	Venison Jerky	*p* Value
L*	30.9 ± 7.5 ^ab^	30.4 ± 4.7 ^a^	34.4 ± 6.8 ^b^	28.1 ± 2.0 ^a^	34.6 ± 5.9 ^b^	30.9 ± 5.7 ^ab^	0.040
a*	6.57 ± 3.8 ^a^	8.15 ± 3.37 ^ab^	9.77 ± 4.72 ^b^	5.01 ± 1.60 ^a^	10.04 ± 3.64 ^b^	7.52 ± 3.24 ^ab^	0.014
b*	10.41 ± 7.12	10.22 ± 5.78	13.69 ± 8.45	5.69 ± 1.26	13.35 ± 6.11	8.25 ± 4.90	0.051
C*	12.4 ± 7.9 ^ab^	13.3 ± 6.3 ^ab^	17.1 ± 9.2 ^b^	7.64 ± 1.76 ^a^	16.82 ± 6.83 ^b^	11.2 ± 5.7 ^ab^	0.029
h°	56.5 ± 6.8	49.4 ± 10.6	52.4 ± 12.2	49.1 ± 7.1	51.1 ± 8.4	45.6 ± 7.0	0.130
WBSF	74.9 ± 34.0	83.9 ± 38.0	73.5 ± 44.3	52.8 ± 37.0	55.2 ± 24.5	62.6 ± 24.5	0.064
Colour	6.43 ± 1.50 ^bc^	6.84 ± 1.71 ^bc^	4.48 ± 1.43 ^a^	4.75 ± 1.31 ^ab^	4.55 ± 0.99 ^a^	7.56 ± 1.06 ^c^	<0.001
Odour	5.62 ± 1.96	6.16 ± 1.88	5.85 ± 1.93	6.47 ± 0.38	6.20 ± 0.79	5.96 ± 1.94	0.413
Taste	5.27 ± 2.19	5.50 ± 2.19	5.13 ± 2.15	6.87 ± 0.68	5.27 ± 0.92	4.96 ± 2.14	0.238
Saltiness	5.38 ± 1.58	5.06 ± 1.80	5.60 ± 1.69	6.34 ± 0.67	5.17 ± 1.10	5.74 ± 1.26	0.230
Tenderness	6.19 ± 1.94 ^a^	4.57 ± 2.12 ^b^	5.36 ± 1.87 ^ab^	4.51 ± 1.39 ^ab^	4.06 ± 1.28 ^b^	3.70 ± 1.89 ^b^	<0.001
Overall acceptance	5.24 ± 2.16	5.26 ± 2.10	5.26 ± 1.96	6.35 ± 0.72	4.87 ± 0.73	4.67 ± 1.98	0.221

^a,b,c^ Different superscripts represent statistically significant differences between values within each row.

## Data Availability

The original contributions presented in this study are included in the article and Supplementary Materials. Further inquiries can be directed to the corresponding author.

## Supplementary Materials

The following supporting information can be downloaded at: https://www.mdpi.com/article/10.3390/foods14213792/s1.
