# Supplementary material for: Jerky and Biltong from the Czech Retail Market: Microbial Quality, Chemical Composition, and Other Quality Characteristics
Source: Foods. 2025 Nov 5;14(21):3792. doi: 10.3390/foods14213792 (PMC12607738; doi:10.3390/foods14213792)
Supplement: Supplementary file 1 [file foods-14-03792-s001.zip › foods-3942959-supplementary.pdf]

Microbiological analysis

**Sample number**	**Sample**	**Batch**	**CPM log**	***Enterobact* log**	**BMK log**	***B. cereus log***	**Clostridium**	**Mould log**
**1**	beef	1	3,88	<1	2,45	<1,7	<1,7	<2
**2**	beef	1	1,48	<1	<1,7	<1,7	<1,7	<2
**3**	beef	1	4,45	<1	2,79	<1,7	<1,7	<2
**4**	beef	1	3,20	<1	3,32	<1,7	<1,7	<2
**5**	turkey	1	2,53	<1	2,30	<1,7	<1,7	<2
**6**	turkey	1	<1	<1	<1,7	<1,7	<1,7	<2
**7**	turkey	1	3,91	<1	2,78	<1,7	<1,7	<2
**8**	beef	1	2,51	<1	2,54	<1,7	<1,7	<2
**9**	beef	1	2,83	<1	2,36	2,40	<1,7	<2
**10**	beef	1	<1	<1	2,45	<1,7	<1,7	<2
**11**	pork	1	3,28	<1	2,48	2,00	<1,7	<2
**12**	pork	1	1,78	<1	<1,7	<1,7	<1,7	<2
**13**	turkey	1	<1	<1	<1,7	<1,7	<1,7	<2
**14**	venison	1	<1	<1	<1,7	<1,7	<1,7	2,48
**15**	venison	1	2,49	<1	1,88	2,30	<1,7	<2
**16**	beef	1	2,18	<1	<1,7	<1,7	<1,7	<2
**17**	beef	1	4,40	<1	2,85	<1,7	<1,7	<2
**18**	beef	1	5,08	<1	3,95	<1,7	<1,7	<2
**19**	turkey	1	5,36	1,60	4,08	<1,7	<1,7	<2
**20**	beef	2	3,85	1,18	3,08	<1,7	<1,7	<2
**21**	beef	2	2,56	<1	2,58	<1,7	<1,7	<2
**22**	turkey	2	3,23	<1	2,95	<1,7	<1,7	<2
**23**	turkey	2	2,54	<1	2,11	<1,7	<1,7	<2
**24**	turkey	2	4,26	<1	2,70	<1,7	<1,7	<2
**25**	venison	2	1,70	<1	2,40	<1,7	<1,7	<2
**26**	beef	1	3,08	<1	<1,7	1,70	<1,7	<2
**27**	beef	1	2,34	<1	<1,7	<1,7	<1,7	<2
**28**	beef	1	5,32	<1	2,65	2,65	<1,7	<2
**29**	beef	2	1,40	<1	<1,7	<1,7	<1,7	<2
**30**	turkey	1	1,00	<1	<1,7	<1,7	<1,7	<2
**31**	turkey	2	1,18	<1	<1,7	<1,7	<1,7	<2
**32**	pork	1	<1	<1	<1,7	<1,7	<1,7	<2
**33**	pork	2	3,04	<1	<1,7	<1,7	<1,7	<2
**34**	venison	1	4,81	<1	4,64	1,70	<1,7	<2
**35**	chicken	1	2,00	<1	<1,7	<1,7	<1,7	<2
**36**	beef	1	2,20	<1	<1,7	<1,7	<1,7	<2

**37**	beef	1	1,85	<1	<1,7	<1,7	<1,7	<2
**38**	turkey	1	3,30	<1	2,40	1,70	<1,7	2,48
**39**	turkey	1	3,15	<1	<1,7	<1,7	<1,7	<2
**40**	pork	1	4,41	<1	<1,7	<1,7	<1,7	<2
**41**	venison	1	5,04	<1	3,38	1,70	<1,7	2,85
**42**	chicken	2	3,08	<1	2,26	<1,7	<1,7	2,30
**43**	venison	2	2,91	<1	2,97	<1,7	<1,7	<2
**44**	turkey	2	1,88	<1	<1,7	<1,7	<1,7	<2
**45**	biltong	1	4,30	<1	2,84	<1,7	<1,7	<2
**46**	pork	1	3,57	<1	2,90	<1,7	<1,7	<2
**47**	pork	2	4,08	<1	<1,7	<1,7	<1,7	<2
**48**	beef	2	2,91	<1	2,54	2,60	<1,7	<2
**49**	beef	2	5,20	<1	4,51	<1,7	<1,7	<2
**50**	beef	2	2,81	<1	2,26	<1,7	<1,7	<2
**51**	turkey	2	4,89	<1	2,99	<1,7	<1,7	<2
**52**	turkey	2	2,82	<1	<1,7	<1,7	<1,7	<2
**53**	venison	2	1,74	<1	2,18	<1,7	<1,7	<2
**54**	biltong	1	2,34	<1	<1,7	1,70	<1,7	<2
**55**	biltong	2	4,04	<1	3,15	<1,7	<1,7	<2
**56**	venison	2	4,76	<1	3,59	<1,7	<1,7	<2
**57**	beef	2	2,32	<1	<1,7	<1,7	<1,7	<2
**58**	turkey	2	4,18	<1	3,04	2,18	<1,7	<2
**59**	biltong	1	2,56	<1	2,88	<1,7	<1,7	<2
**60**	biltong	1	5,20	<1	3,98	1,70	<1,7	<2
**61**	biltong	1	7,62	<1	6,72	<1,7	<1,7	5,38
**62**	beef	1	2,40	<1	2,26	<1,7	<1,7	<2
**63**	beef	1	8,34	3,04	7,54	<1,7	<1,7	<2
**64**	pork	1	8,28	<1	7,11	<1,7	<1,7	<2
**65**	chicken	1	9,79	2,36	7,32	<1,7	<1,7	<2
**66**	beef	2	2,92	<1	<1,7	<1,7	<1,7	<2
**67**	beef	2	5,08	<1	1,40	<1,7	<1,7	<2
**68**	beef	2	2,48	<1	<1,7	<1,7	<1,7	<2
**69**	beef	2	2,95	<1	2,52	<1,7	<1,7	<2
**70**	beef	2	6,98	1,48	6,92	<1,7	<1,7	<2
**71**	turkey	1	7,26	2,04	6,95	<1,7	<1,7	<2
**72**	turkey	2	7,18	1,18	7,23	<1,7	<1,7	<2
**73**	turkey	1	<1	<1	2,00	<1,7	<1,7	<2
**74**	pork	2	1,54	<1	<1,7	<1,7	<1,7	<2
**75**	pork	2	3,28	<1	4,40	<1,7	<1,7	<2
**76**	pork	1	3,28	<1	<1,7	<1,7	<1,7	<2
**77**	chicken	2	7,30	3,45	7,18	<1,7	<1,7	<2
**78**	biltong	2	1,85	<1	1,88	<1,7	<1,7	<2

**79**	biltong	1	5,54	<1	5,63	<1,7	<1,7	<2
**80**	biltong	2	3,36	<1	3,82	<1,7	<1,7	<2
**81**	biltong	2	7,59	2,61	6,23	<1,7	<1,7	4,28
**82**	biltong	2	5,87	<1	5,41	<1,7	<1,7	4,28
**83**	beef	1	4,30	<1	2,85	<1,7	<1,7	<2
**84**	pork	2	3,46	<1	2,52	<1,7	<1,7	<2
**85**	chicken	1	3,56	<1	2,65	<1,7	<1,7	<2
**86**	beef	1	3,00	<1	<1,7	<1,7	<1,7	<2
**87**	biltong	1	6,20	<1	5,73	<1,7	<1,7	<2
**88**	turkey	2	1,78	<1	<1,7	<1,7	<1,7	<2
**89**	chicken	2	4,40	<1	<1,7	<1,7	<1,7	<2
**90**	beef	2	4,32	<1	<1,7	<1,7	<1,7	2,78
**91**	biltong	2	3,49	<1	<1,7	<1,7	<1,7	<2
**92**	biltong	2	5,66	<1	5,86	<1,7	<1,7	<2

Physicochemical analysis

**Sample number**	**Sample**	**Batch**	**pH**	**a_w**	**Totel protein**	**Pure protein**	**Collagen**	**Fat**	**Dry matter**	**Ash**	**TBARS**	**NaCl**
**1**	Beef	1	5,54	0,828	47,82	39,65	2,14	14,67	70,50	6,12	1,70	3,14
**2**	Beef	2	5,47	0,839	49,79	43,38	1,82	6,84	66,63	6,25	1,20	3,27
**3**	Beef	1	5,69	0,735	59,17	50,41	3,21	8,32	76,75	7,04	1,47	3,64
**4**	Beef	2	5,53	0,682	60,71	50,85	1,74	8,66	79,90	6,97	1,55	3,92
**5**	Beef	1	5,59	0,766	47,22	41,21	2,94	10,37	71,84	6,50	2,33	3,50
**6**	Beef	2	5,22	0,716	47,45	43,51	1,99	6,57	75,03	6,57	1,59	3,48
**7**	Beef	1	5,45	0,773	39,26	34,23	1,16	7,54	69,59	7,95	1,79	4,27
**8**	Beef	2	5,22	0,717	40,11	33,65	1,09	2,06	73,19	3,35	18,39	4,96
**9**	Beef	1	5,54	0,493	33,71	29,63	1,40	1,92	88,79	5,79	29,78	3,49
**10**	Beef	2	5,58	0,550	33,18	29,70	1,43	2,32	88,41	5,21	62,74	3,24
**11**	Beef	1	5,76	0,874	48,37	43,21	2,14	6,50	62,53	5,67	3,78	3,35
**12**	Beef	2	5,67	0,869	47,25	41,87	2,04	6,51	63,94	6,12	3,59	3,26
**13**	Beef	1	5,30	0,616	61,90	53,29	2,25	5,64	82,36	8,54	3,20	4,53
**14**	Beef	2	5,48	0,662	61,62	45,16	2,43	8,87	82,45	6,52	3,43	3,63
**15**	Beef	1	5,29	0,808	34,45	30,39	1,53	4,47	70,87	4,70	6,86	2,88
**16**	Beef	2	5,48	0,776	34,91	29,69	1,92	3,40	71,55	4,91	10,69	2,96
**17**	Beef	1	5,52	0,771	54,33	48,51	2,50	9,54	75,16	5,95	1,94	3,83
**18**	Beef	2	5,78	0,733	57,40	50,58	2,17	6,07	73,80	6,34	1,28	3,77
**19**	Beef	1	5,15	0,775	50,39	38,86	1,25	7,66	71,79	6,31	2,80	3,47
**20**	Beef	2	5,33	0,611	55,54	46,74	1,31	10,52	81,31	7,64	1,20	3,68
**21**	Beef	1	5,42	0,752	58,64	51,45	1,48	6,92	75,09	6,85	2,77	3,90
**22**	Beef	2	5,97	0,708	53,73	51,62	2,99	8,61	76,98	6,86	1,52	3,82
**23**	Beef	1	5,70	0,873	53,97	48,30	3,32	7,49	67,82	4,49	2,26	2,22

**24**	Beef	2	5,56	0,890	49,70	44,16	2,15	8,82	65,63	3,95	2,54	2,01
**25**	Beef	1	5,69	0,656	67,88	57,78	1,20	4,78	80,64	7,52	1,09	3,49
**26**	Beef	2	5,84	0,693	60,16	51,44	2,46	8,39	78,19	7,16	1,56	3,70
**27**	Beef	1	5,49	0,594	60,43	53,57	2,70	9,13	83,60	7,90	5,07	3,69
**28**	Beef	2	5,62	0,591	62,35	55,01	1,50	2,92	84,28	7,86	9,77	3,46
**29**	Beef	1	4,87	0,729	40,16	32,84	1,40	2,65	71,60	4,54	11,07	2,57
**30**	Beef	2	4,92	0,721	36,91	32,05	1,28	4,68	71,63	4,64	9,25	2,72
**31**	Beef	1	4,86	0,729	39,14	37,93	1,64	3,42	71,79	8,32	2,64	4,17
**32**	Beef	2	5,49	0,724	54,20	47,98	1,81	3,10	71,59	8,02	3,94	4,58
**33**	Pork	1	5,32	0,770	47,62	40,57	1,15	6,99	69,32	6,33	1,35	3,40
**34**	Pork	2	5,72	0,871	43,96	42,72	1,91	8,17	63,33	5,47	4,97	3,76
**35**	Pork	1	5,41	0,868	46,62	41,57	1,57	9,67	63,63	5,73	4,32	3,21
**36**	Pork	2	5,54	0,845	47,84	42,58	1,26	14,07	67,67	6,04	3,42	3,70
**37**	Pork	1	5,75	0,825	55,09	49,27	0,61	10,82	70,45	4,50	3,90	2,27
**38**	Pork	2	5,09	0,758	59,06	51,37	0,98	5,85	74,50	5,30	0,81	2,60
**39**	Pork	1	5,44	0,840	53,06	45,99	1,26	5,15	67,30	6,18	0,78	3,30
**40**	Pork	2	5,48	0,842	55,28	48,83	0,82	4,01	69,45	6,36	0,94	3,26
**41**	Pork	1	5,22	0,613	56,61	42,82	0,90	11,89	85,37	9,18	1,29	4,46
**42**	Pork	2	4,87	0,593	60,27	50,57	1,15	11,96	85,08	6,63	0,66	3,98
**43**	Pork	1	5,32	0,561	66,21	58,28	1,14	3,29	84,77	8,49	2,18	3,94
**44**	Pork	2	5,51	0,661	58,74	51,54	1,05	5,61	78,66	7,95	1,28	3,75
**45**	Turkey	1	5,70	0,860	45,31	41,57	1,43	3,15	67,20	6,02	0,51	3,31
**46**	Turkey	2	5,65	0,851	52,24	43,22	0,83	2,50	62,99	6,19	0,47	3,23
**47**	Turkey	1	5,71	0,737	62,99	40,74	1,53	3,66	74,70	6,91	0,55	4,08
**48**	Turkey	2	5,67	0,760	61,39	52,35	1,97	2,77	72,01	7,11	0,62	4,28
**49**	Turkey	1	5,56	0,748	48,85	39,30	0,74	4,47	71,83	6,04	1,24	3,48
**50**	Turkey	2	5,36	0,719	51,46	43,48	0,82	4,47	72,47	6,93	1,21	4,17
**51**	Turkey	1	5,74	0,867	49,29	41,71	1,20	5,86	62,70	5,64	3,72	3,28
**52**	Turkey	2	5,73	0,856	46,74	41,32	1,25	9,32	64,06	5,48	3,60	3,38
**53**	Turkey	1	5,49	0,619	65,14	56,00	1,01	3,90	81,43	8,81	1,05	4,27
**54**	Turkey	2	5,43	0,642	67,26	56,73	0,96	3,09	81,99	8,12	1,01	4,27
**55**	Turkey	1	5,81	0,776	54,44	52,25	1,57	2,50	72,16	6,70	10,92	3,85
**56**	Turkey	2	5,70	0,676	66,23	58,03	0,82	2,79	77,53	7,65	0,76	4,22
**57**	Turkey	1	5,29	0,687	42,82	37,59	0,81	3,22	75,91	8,04	8,34	4,69
**58**	Turkey	2	5,29	0,670	44,02	35,95	0,83	3,64	75,91	7,97	21,89	4,25
**59**	Turkey	1	5,90	0,880	54,79	48,06	1,18	6,02	67,56	4,25	1,36	1,97

**60**	Turkey	2	5,75	0,871	55,63	48,73	0,89	6,42	68,79	3,82	1,63	2,11
**61**	Turkey	1	4,39	0,595	68,59	58,53	1,10	2,30	83,45	7,19	0,94	3,28
**62**	Turkey	2	4,38	0,568	69,89	54,23	1,14	2,07	84,84	7,76	1,05	3,56
**63**	Turkey	1	6,03	0,710	64,98	55,46	1,39	2,89	75,41	7,17	1,09	4,25
**64**	Turkey	2	5,94	0,705	64,63	53,86	1,22	3,45	75,35	7,10	0,86	3,99
**65**	Venison	1	5,47	0,825	53,24	47,55	2,68	6,20	68,13	6,18	5,72	3,58
**66**	Venison	2	5,40	0,849	48,15	42,63	2,11	9,26	66,28	5,69	1,90	3,22
**67**	Venison	1	5,24	0,758	53,02	48,38	1,24	3,18	72,68	6,80	1,32	4,05
**68**	Venison	2	5,40	0,774	59,08	51,29	1,03	3,47	72,72	6,44	1,44	3,53
**69**	Venison	1	5,73	0,693	65,75	55,91	0,88	3,77	78,65	6,80	1,63	4,10
**70**	Venison	2	5,57	0,746	61,87	51,07	1,06	2,94	74,31	6,71	1,55	3,66
**71**	Venison	1	5,27	0,762	57,09	49,23	1,09	4,27	73,10	6,58	1,78	3,55
**72**	Venison	2	5,74	0,718	59,66	50,20	1,66	4,55	76,14	6,32	1,91	3,35
**73**	Chicken	1	5,72	0,851	50,65	45,50	0,64	4,87	63,71	6,04	0,97	3,39
**74**	Chicken	2	5,72	0,827	44,56	42,34	0,97	3,60	65,67	6,63	0,86	3,42
**75**	Chicken	1	5,49	0,549	68,89	57,76	1,17	4,23	85,24	7,68	1,05	3,51
**76**	Chicken	2	5,81	0,595	66,41	55,24	1,05	4,26	82,84	7,81	0,39	3,51
**77**	Chicken	1	5,86	0,614	63,11	52,93	0,95	5,80	80,69	8,30	1,16	4,26
**78**	Chicken	2	5,96	0,655	60,41	51,88	1,09	6,11	79,07	8,03	1,09	4,31
**79**	Biltong	1	5,09	0,773	53,02	46,50	1,46	3,79	70,00	5,89	2,02	3,22
**80**	Biltong	2	4,99	0,658	54,12	46,39	1,26	2,81	77,17	6,47	1,79	3,58
**81**	Biltong	1	4,89	0,822	38,43	31,59	1,69	5,18	60,90	7,14	1,28	4,25
**82**	Biltong	2	5,33	0,802	40,62	31,39	2,82	4,70	60,67	7,54	2,41	4,58
**83**	Biltong	1	5,09	0,759	51,52	45,04	2,87	5,93	69,63	7,66	1,52	3,90
**84**	Biltong	2	5,36	0,757	51,52	44,69	2,52	7,66	70,34	7,81	1,64	3,92
**85**	Biltong	1	5,13	0,808	55,11	48,64	1,90	6,85	70,22	5,30	2,38	2,84
**86**	Biltong	2	5,12	0,866	49,70	41,11	1,57	12,22	62,89	3,55	1,24	1,66
**87**	Biltong	1	5,32	0,608	63,57	56,21	2,49	8,12	82,43	6,94	6,84	3,39
**88**	Biltong	2	5,64	0,594	58,24	51,79	3,40	16,43	85,38	6,23	3,19	3,25

**89**	Bilto ng	1	5,44	0,805	42,51	37,90	1,01	3,91	63,97	5,76	4,66	3,49
**90**	Bilto ng	2	5,50	0,852	43,44	37,65	2,82	12,43	58,18	5,80	3,74	3,42
**91**	Bilto ng	1	5,12	0,784	43,92	40,04	2,28	7,25	66,13	7,48	2,14	4,07
**92**	Bilto ng	2	5,37	0,798	50,99	43,87	2,70	4,63	66,08	6,91	2,52	4,01

Color and texture analysis

**Sample number**	**Sample**	**Batch**	**L*(D65)**	**a*(D65)**	**b*(D65)**	**C*(D65)**	**h°(D65)**	**WBSF**
**1**	Beef	1	28,09	4,44	4,98	6,68	48,28	151,99
**2**	Beef	2	26,36	4,70	3,41	5,81	35,99	73,59
**3**	Beef	1	27,30	5,67	5,58	7,95	44,57	113,82
**4**	Beef	2	27,17	4,56	4,99	6,75	47,58	132,54
**5**	Beef	1	27,46	5,29	5,92	7,94	48,24	106,54
**6**	Beef	2	26,93	4,53	6,44	7,87	54,87	121,74
**7**	Beef	1	27,67	10,57	6,51	12,41	31,64	47,92
**8**	Beef	2	25,53	6,88	3,21	7,59	24,99	33,05
**9**	Beef	1	31,83	13,54	19,96	24,12	55,85	152,90
**10**	Beef	2	34,55	15,28	26,61	30,68	60,13	99,02
**11**	Beef	1	27,83	8,88	5,38	10,38	31,23	51,69
**12**	Beef	2	26,94	6,50	4,98	8,19	37,45	39,91
**13**	Beef	1	33,72	4,76	10,46	11,49	65,52	72,47
**14**	Beef	2	29,99	4,60	7,08	8,45	56,97	105,75
**15**	Beef	1	25,88	6,45	4,98	8,15	37,69	41,83
**16**	Beef	2	26,57	7,78	6,03	9,84	37,76	40,54
**17**	Beef	1	28,38	6,43	4,80	8,03	36,73	141,50
**18**	Beef	2	29,40	5,25	6,54	8,38	51,27	105,47
**19**	Beef	1	22,79	7,14	6,92	9,94	44,12	54,03
**20**	Beef	2	29,04	7,91	10,12	12,84	51,98	52,37
**21**	Beef	1	26,07	3,23	3,88	5,05	50,23	122,93
**22**	Beef	2	28,23	4,40	5,44	7,00	50,98	106,51
**23**	Beef	1	24,52	5,45	4,67	7,18	40,62	81,60
**24**	Beef	2	23,57	6,76	5,21	8,54	37,61	52,59
**25**	Beef	1	29,51	4,11	6,08	7,34	55,97	77,35
**26**	Beef	2	27,53	5,05	4,30	6,63	40,45	122,84
**27**	Beef	1	29,21	4,87	8,27	9,60	59,50	126,93
**28**	Beef	2	29,28	4,26	7,72	8,82	61,08	161,08
**29**	Beef	1	22,46	2,26	3,53	4,19	57,39	58,62
**30**	Beef	2	23,08	3,10	5,29	6,13	59,59	51,39
**31**	Beef	1	27,48	3,34	5,91	6,79	60,54	184,67
**32**	Beef	2	25,16	3,96	6,84	7,91	59,93	60,27

**33**	Pork	1	32,14	12,38	14,13	18,78	48,78	76,93
**34**	Pork	2	34,26	11,47	12,98	17,65	49,27	73,58
**35**	Pork	1	41,11	10,91	19,53	22,37	60,80	28,00
**36**	Pork	2	39,62	10,46	12,64	16,41	50,38	34,01
**37**	Pork	1	38,49	12,63	18,47	22,37	55,63	30,46
**38**	Pork	2	38,03	16,24	19,88	25,67	50,75	38,12
**39**	Pork	1	35,98	11,11	12,16	16,47	47,60	72,41
**40**	Pork	2	31,78	13,53	10,04	16,85	36,57	69,58
**41**	Pork	1	46,80	11,85	24,47	27,19	64,16	144,57
**42**	Pork	2	35,13	11,93	16,07	20,02	53,42	97,40
**43**	Pork	1	32,29	12,13	15,78	19,90	52,45	88,87
**44**	Pork	2	32,70	11,30	17,58	20,90	57,28	100,43
**45**	Turkey	1	36,26	10,66	14,29	17,83	53,26	59,15
**46**	Turkey	2	33,70	10,94	11,72	16,03	46,97	43,75
**47**	Turkey	1	34,28	10,20	13,72	17,09	53,38	38,48
**48**	Turkey	2	41,52	11,23	18,14	21,33	58,22	78,02
**49**	Turkey	1	39,54	9,29	17,13	19,48	61,53	43,17
**50**	Turkey	2	37,04	11,73	18,24	21,68	57,24	55,20
**51**	Turkey	1	38,87	15,24	17,21	22,99	48,47	35,79
**52**	Turkey	2	40,07	13,17	18,01	22,31	53,82	34,70
**53**	Turkey	1	38,47	12,40	19,25	22,90	57,21	75,53
**54**	Turkey	2	43,08	12,91	23,19	26,54	60,89	90,99
**55**	Turkey	1	39,58	7,85	18,22	19,83	66,69	46,60
**56**	Turkey	2	37,68	13,86	14,49	20,05	46,28	71,64
**57**	Turkey	1	30,52	12,77	12,57	17,92	44,57	52,22
**58**	Turkey	2	32,82	12,71	15,45	20,01	50,55	48,92
**59**	Turkey	1	35,20	10,70	16,35	19,54	56,79	73,67
**60**	Turkey	2	39,67	9,52	15,48	18,17	58,42	64,76
**61**	Turkey	1	34,35	13,49	19,92	24,06	55,89	95,10
**62**	Turkey	2	40,33	12,34	20,39	23,83	58,81	74,42
**63**	Turkey	1	42,71	17,31	22,22	28,17	52,07	59,91
**64**	Turkey	2	33,70	11,59	12,16	16,79	46,38	68,23
**65**	Venison	1	28,82	7,41	6,83	10,08	42,65	55,27
**66**	Venison	2	27,85	5,19	3,81	6,44	36,30	31,18
**67**	Venison	1	27,82	5,02	4,74	6,90	43,32	80,74
**68**	Venison	2	26,55	5,28	3,67	6,44	34,82	47,90
**69**	Venison	1	28,91	2,54	5,47	6,03	65,09	108,45
**70**	Venison	2	29,71	4,79	4,87	6,83	45,45	63,09
**71**	Venison	1	26,84	2,34	2,31	3,29	44,55	59,63
**72**	Venison	2	27,21	3,64	3,76	5,24	45,96	68,73
**73**	Chicken	1	41,54	10,55	16,79	19,82	57,86	38,10
**74**	Chicken	2	30,60	8,46	9,17	12,48	47,30	54,02
**75**	Chicken	1	47,35	13,60	27,00	30,23	63,27	59,44
**76**	Chicken	2	38,29	10,67	16,33	19,50	56,83	59,95
**77**	Chicken	1	38,40	11,18	19,13	22,15	59,70	81,70

**78**	Chicken	2	45,32	11,82	25,36	27,98	65,00	74,35
**79**	Biltong	1	32,56	9,41	11,55	14,90	50,82	20,74
**80**	Biltong	2	27,76	7,67	7,70	10,87	45,11	31,98
**81**	Biltong	1	28,06	5,89	5,86	8,31	44,86	35,78
**82**	Biltong	2	26,98	5,66	6,35	8,51	48,30	24,39
**83**	Biltong	1	27,14	9,74	6,36	11,63	33,13	42,66
**84**	Biltong	2	25,39	8,08	3,68	8,88	24,49	42,70
**85**	Biltong	1	29,33	5,94	9,91	11,56	59,06	63,28
**86**	Biltong	2	24,03	5,57	8,41	10,09	56,50	27,11
**87**	Biltong	1	30,87	4,59	6,58	8,02	55,10	69,42
**88**	Biltong	2	33,52	3,51	8,74	9,42	68,13	142,17
**89**	Biltong	1	28,13	3,80	5,49	6,68	55,32	38,78
**90**	Biltong	2	22,84	2,78	4,10	4,95	55,91	55,90
**91**	Biltong	1	31,11	4,79	7,52	8,92	57,51	27,32
**92**	Biltong	2	31,00	3,73	8,03	8,85	65,06	30,20

Sensory analysis

**Sample number**	**Sample**	**Batch**	**Color**	**Odor**	**Tatse**	**Saltiness**	**Tendernes s**	**Overall acceptanc e**
**1**	Beef	1	7,50	6,75	6,88	4,13	4,13	7,13
**2**	Beef	2	7,14	7,29	7,00	6,14	4,00	7,29
**3**	Beef	1	6,63	5,88	6,00	5,38	2,13	5,63
**4**	Beef	2	6,86	6,86	7,71	5,86	3,86	7,00
**5**	Beef	1	7,50	6,38	6,38	5,38	4,13	5,75
**6**	Beef	2	6,86	5,86	5,86	4,71	4,86	5,71
**7**	Beef	1	5,43	7,00	4,71	4,71	7,71	5,43
**8**	Beef	2	5,57	6,14	4,71	3,57	6,71	4,43
**9**	Beef	1	3,14	5,00	3,86	3,00	4,86	3,43
**10**	Beef	2	3,33	5,50	4,83	2,83	5,50	3,50
**11**	Beef	1	5,57	7,43	6,57	5,86	4,00	6,43
**12**	Beef	2	7,43	6,57	6,57	4,71	4,57	6,14
**13**	Beef	1	6,43	5,00	4,86	4,86	3,14	4,29
**14**	Beef	2	7,57	5,57	4,67	6,14	3,29	4,71
**15**	Beef	1	7,17	6,33	3,00	3,00	5,17	3,17
**16**	Beef	2	6,00	5,00	3,60	2,60	7,20	3,40
**17**	Beef	1	7,50	6,17	6,40	5,50	3,67	5,67
**18**	Beef	2	6,67	7,00	7,00	7,67	5,00	6,00
**19**	Beef	1	6,57	6,57	5,00	5,43	6,14	5,29
**20**	Beef	2	6,71	6,57	6,57	6,71	6,57	6,71
**21**	Beef	1						
**22**	Beef	2	7,63	6,25	6,38	5,63	2,63	5,25
**23**	Beef	1	8,14	5,71	6,43	5,71	4,57	6,43

**24**	Beef	2	8,17	6,33	6,67	5,00	5,33	5,67
**25**	Beef	1	7,57	6,29	5,14	6,29	3,71	5,29
**26**	Beef	2	7,80	7,00	4,80	5,00	2,60	5,20
**27**	Beef	1	6,33	5,00	5,67	6,33	6,00	5,00
**28**	Beef	2						
**29**	Beef	1	8,57	5,43	2,86	4,29	6,00	2,86
**30**	Beef	2	7,43	4,86	2,86	4,71	4,29	2,29
**31**	Beef	1	7,71	6,43	5,57	6,86	3,71	5,71
**32**	Beef	2	7,67	5,00	7,00	5,67	4,00	6,67
**33**	Pork	1	6,43	5,57	4,86	5,00	4,43	4,57
**34**	Pork	2						
**35**	Pork	1	3,86	6,71	5,43	6,00	5,43	5,86
**36**	Pork	2	3,50	5,00	4,17	6,17	5,17	4,60
**37**	Pork	1	4,67	4,33	3,33	4,33	6,50	3,33
**38**	Pork	2	4,50	3,20	2,40	4,00	5,80	3,00
**39**	Pork	1	5,29	7,29	7,00	6,14	5,57	7,29
**40**	Pork	2	5,00	7,83	6,80	6,00	3,17	6,67
**41**	Pork	1	4,29	5,43	5,29	6,14	5,86	4,71
**42**	Pork	2	5,00	6,67	7,00	5,33	5,33	6,33
**43**	Pork	1	5,67	5,67	5,33	6,00	4,67	5,33
**44**	Pork	2						
**45**	Turkey	1	5,13	7,38	6,63	5,63	4,63	6,75
**46**	Turkey	2	5,00	6,00	6,13	5,38	5,50	5,25
**47**	Turkey	1	5,13	7,13	5,25	5,50	2,88	5,13
**48**	Turkey	2	4,43	6,71	6,50	6,43	4,29	5,14
**49**	Turkey	1	3,13	4,88	4,88	4,63	4,25	4,88
**50**	Turkey	2	4,14	5,86	5,71	4,57	4,29	4,71
**51**	Turkey	1	4,71	6,57	5,29	4,71	2,71	5,29
**52**	Turkey	2	4,00	6,50	4,50	4,33	4,50	4,83
**53**	Turkey	1	4,00	5,71	5,00	6,86	6,29	4,57
**54**	Turkey	2	3,57	5,71	6,00	6,71	5,14	4,57
**55**	Turkey	1	3,63	6,25	5,25	5,63	3,25	5,25
**56**	Turkey	2	6,88	5,75	4,75	5,63	2,38	4,13
**57**	Turkey	1	5,50	5,38	3,75	3,88	4,75	3,75
**58**	Turkey	2	4,86	5,14	3,43	3,43	5,14	4,00
**59**	Turkey	1	4,29	5,71	4,71	4,14	3,86	4,71
**60**	Turkey	2	3,00	6,17	5,33	3,67	5,33	4,67
**61**	Turkey	1						
**62**	Turkey	2						
**63**	Turkey	1	4,60	7,80	6,80	7,00	2,20	6,00
**64**	Turkey	2	6,00	7,00	5,00	5,00	1,67	4,00
**65**	Venison	1	6,71	7,86	7,29	5,14	4,00	6,86
**66**	Venison	2	6,86	5,14	3,71	5,00	5,14	4,29
**67**	Venison	1	7,14	5,29	4,57	6,00	3,86	4,71
**68**	Venison	2	7,33	6,67	4,67	4,83	3,33	5,00

**69**	Venison	1	7,83	5,50	5,00	5,67	2,50	4,33
**70**	Venison	2	8,14	5,57	5,00	6,43	3,00	4,14
**71**	Venison	1	8,29	6,29	4,14	6,43	3,00	3,71
**72**	Venison	2	8,14	5,43	5,29	6,29	4,57	4,29
**73**	Chicken	1	4,67	6,33	7,17	5,50	5,83	6,83
**74**	Chicken	2	6,63	6,88	7,13	6,38	4,25	6,63
**75**	Chicken	1	5,33	5,00	6,00	6,33	5,00	5,67
**76**	Chicken	2						
**77**	Chicken	1	4,00	6,67	7,33	6,33	2,67	6,67
**78**	Chicken	2	3,71	6,00	5,86	7,14	5,29	5,29
**79**	Biltong	1	5,50	5,63	6,25	4,75	7,13	6,50
**80**	Biltong	2	7,33	6,67	5,83	4,67	7,00	6,17
**81**	Biltong	1	7,33	6,50	4,00	4,67	6,00	4,50
**82**	Biltong	2	7,00	6,00	6,67	5,00	6,00	6,00
**83**	Biltong	1	5,14	6,57	6,57	6,29	7,00	5,86
**84**	Biltong	2	5,40	5,20	5,20	5,40	7,00	5,40
**85**	Biltong	1	7,00	5,67	5,67	5,67	6,33	6,00
**86**	Biltong	2	7,43	4,29	2,14	3,57	6,57	2,29
**87**	Biltong	1	6,67	5,00	4,67	5,00	7,33	5,67
**88**	Biltong	2	5,71	3,57	3,86	5,43	3,00	3,71
**89**	Biltong	1	5,86	5,71	5,14	6,00	5,43	4,71
**90**	Biltong	2	6,57	5,71	5,71	5,71	5,71	5,14
**91**	Biltong	1	7,29	6,29	6,29	7,14	6,43	6,14
**92**	Biltong	2	7,00	6,33	7,33	6,00	7,00	7,67

Sensory evaluation was not carried out because the microbiological results were unacceptable.
